# H2A.Z-nucleosomes are stabilized by the superhelicity-dependent DNA binding of the C-terminal tail of the histone variant

**DOI:** 10.1080/19491034.2025.2557113

**Published:** 2025-09-09

**Authors:** Ibtissem Benhamza, Laszlo Imre, Zutao Yu, Peter Nanasi, Pialy Sen, Kata Nora Enyedi, Katalin Goda, György Vamosi, Gabor Szabo

**Affiliations:** a Department of Biophysics and Cell Biology, Faculty of Medicine, University of Debrecen, Debrecen, Hungary; b Yusuf Hamied Department of Chemistry, University of Cambridge, Cambridge, UK; c Department of Organic Chemistry, Institute of Chemistry, Eötvös Loránd University, Budapest, Hungary; d HUN-REN-ELTE, Supported Research Groups, Research Group of Peptide Chemistry, Budapest, Hungary

**Keywords:** H2A.Z, nucleosome stability, superhelicity, C-terminal tail

## Abstract

Using an in situ nucleosome stability assay based on salt extraction, we identified distinct stability features of H2A.Z-containing nucleosomes linked to alternative interactions of the histone variant’s C-terminal tail (Imre et al., Nat. Commun., 2024). In DT40 cells expressing either full-length or C-terminally truncated human H2A.Z1, we show that nucleosome stability is tail-dependent also through the spectacles of intercalator sensitivity, raising the possibility that the tail may bind to DNA in a superhelicity-dependent fashion. Supporting this, fluorescence correlation spectroscopy detected binding of a fluorescent H2A.Z-tail nonapeptide to supercoiled—but not relaxed—plasmid DNA, while a scrambled peptide showed negligible binding. The DNA topology-dependent binding of the unstructured H2A.Z C-terminus, by affecting nucleosome stability, may be of functional significance in various roles of the histone variant, demonstrating the strong interplay between DNA topology and nucleosome stability and exemplifying how it may be exploited by the cell for regulatory purposes.

## Introduction

Nucleosome stability is generally considered as a feature impacting chromatin remodeling, e.g. upon the formation of nucleosome free regions at regulatory sites, and influencing the progression of processive enzymatic functions. The general term ‘stability’ implies chemical forces strengthening cohesive interactions among the histones and between the individual proteins and the DNA. While the DNA is wound around the histone core, it must turn around its axis, forming toroid superhelices involving many histone-DNA chemical bonds constraining the DNA so as to yield an undertwisted overall structure [1]. When the nucleosome is released, the toroid superhelicity becomes manifest in plectonemic supercoils [2]. The stability of these complex nucleoprotein structures can be experimentally challenged both by interfering with the chemical bonds involved in their stabilization and by extending and untwisting the DNA using intercalator compounds that become inserted between adjacent bases sandwich-like. In vivo, posttranslational modifications (PTMs) by themselves and/or by recruiting reader proteins, may set the stage for the chromatin remodeling enzymes to actively move or remove nucleosomes at particular regions of the chromatin. The stability of nucleosomes is often altered also when the canonical core histones are replaced with histone variants, with functional effects [3,4]. However, in the case of H2A.Z, which nonrandomly replaces H2A, the mainly in vitro observations are conflicting and there appears to be no consensus in the field whether the presence of H2A.Z in nucleosomes increases [5–7] or decreases [8–10] nucleosome stability. It has two isoforms, H2A.Z.1 and H2A.Z.2, which differ only by three amino acids and are encoded by separate genes [11,12], and exhibit approximately 60% amino acid sequence identity to canonical H2A [13,14]. The H2A.Z isoforms are essential players in the regulation of transcription, DNA replication, cell cycle, DNA repair, and 3D chromatin structure; they are implicated in embryonic development, cellular differentiation, neurodevelopment and brain function [15–17].

Although the relationship between the result of the modulation of nucleosome stability by different experimental strategies and the resistance of the nucleosomes to enzyme-catalyzed deposition or eviction of particular histones, or remodeling of the entire nucleosome [16,18] and other physiological challenges may be rather indirect, the stability features determined in such experiments are generally deemed to reflect and predict how the same nucleosomes may behave *in vivo*. Among the forces binding the DNA to the histone core, salt bridges between the side-chain guanidinium cation of arginine and the phosphate group of the DNA backbone is thought to be dominant, involving a combination of electrostatic attraction between the charged molecular entities and hydrogen bonds of the guanidinium nitrogens to the phosphate group oxygens [19,20]. These interactions are expected to be modulated both by increasing ionic strength and changing DNA topology.

Using an experimental system where nuclei prepared by nonionic detergent treatment of the embedded, live cells are exposed to treatments disturbing nucleosome integrity, termed ‘Quantitative Imaging of Nuclei after Elution with Salt/Intercalators’ (QINESIn) [21], we have recently observed that the +1 nucleosomes at the transcription start sites (TSSs), recognized by their characteristic PTMs, become less stable when exposed to a concentration series of intercalators, relative to other nucleosomes distinguished by many other PTMs [22]. However, the nucleosomes recognized based on the presence of the H2A.Z histone variant appeared stable, in variance with what may be anticipated based on their frequent association with nucleosomes neighboring the TSSs [23,24]. This seeming discrepancy was dissolved by our observation that the H2A.Z nucleosomes form a heterogeneous landscape in the nucleus, comprising three pools in terms of salt resistance: those present in euchromatin that exhibit stability similar to that of canonical H2A containing nucleosomes, stable ones of the heterochromatin and those attached very strongly to the nuclear lamina [25]. We have also shown that the unstructured C-terminal tail plays a decisive role in determining H2A.Z nucleosome stability and suggested that binding of the tail to the same or a neighboring nucleosome confers increased stability, while its engagement with reader proteins in euchromatin [11,15,26] may prevent these interactions, eliminating this increment. Based on the effects of the treatment of nuclei as well as live cells with the nine amino acid long tail peptide (termed C9), we also concluded that the molecular interactions involving the tail have a large impact on the global chromatin architecture: C9 treatment not only destabilized nucleosomes, as measured through the spectacles of salt sensitivity, but also increased sensitivity to nucleases and altered the whole chromatin landscape [25]. In the same publication, we have also shown in fluorescence correlation spectroscopy (FCS) studies that the carboxyfluorescein-tagged tail peptide (CF-C9) can bind the reconstituted nucleosomes, while its binding to linear, topologically relaxed DNA was barely detectable. Whether the tail peptide can bind to the superhelical DNA of closed topological forms has not been determined, nor was its possible binding to the histone core assessed.

We further investigated the tail-dependence of H2A.Z-nucleosome stability using the other format of the QINESIn assay [21], titrating the sensitivity of nucleosomes to intercalators that alter the superhelical structure of the DNA wrapped around the nucleosomes or linking them to each other. We have compared the intercalator sensitivity of nucleosomes in DT40 cells expressing either the human H2A.Z1 or its C-terminally truncated form; with the endogenous H2A.Z histone genes being knocked out (DKO/Z1 and DKO/ΔC cells¸ respectively [27]). These experiments argued against binding of the tail to the histone core and suggested that H2A.Z may bind via its C-terminal, unstructured tail to DNA in a superhelicity-dependent fashion, what was confirmed in FCS measurements using the tail peptide and plasmid DNA. Implications of these observations in the context of the role of the histone variant in transcriptional regulation, DNA repair and the organization of chromatin domains are discussed.

## Results and discussion

**Figure 1.**
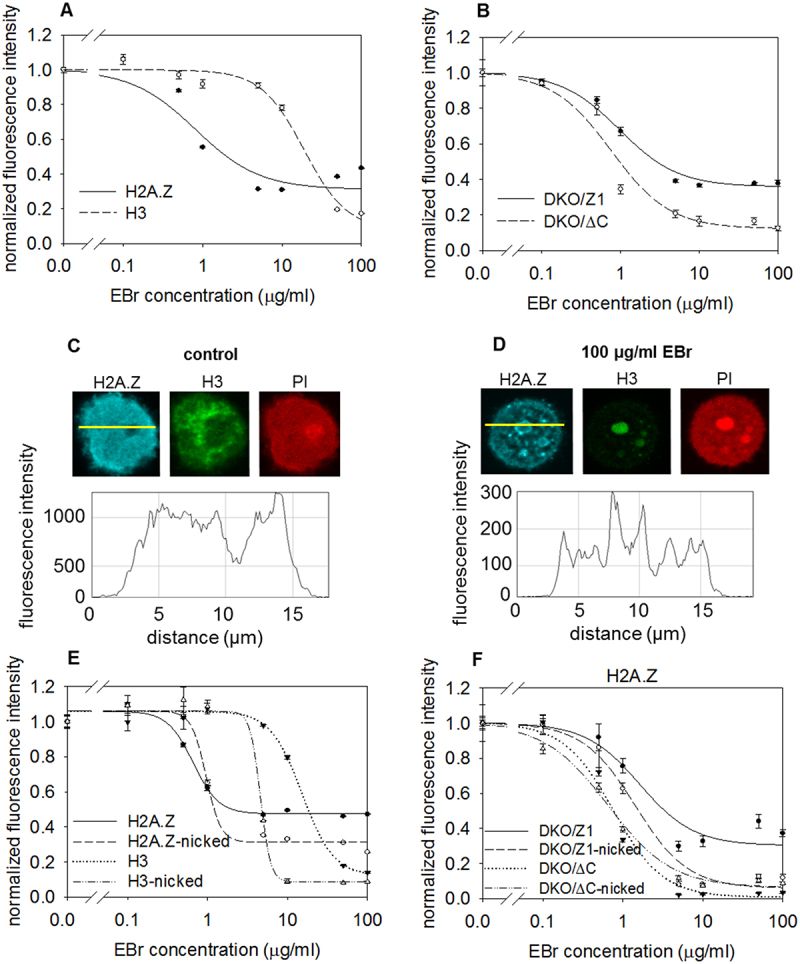
Stability of H2A.Z-containing nucleosomes studied *in situ* by treatment of nuclei with the intercalator dye, EBr. (A) EBr elution profile of H2A.Z compared to GFP-tagged H3 used as an internal control measured in HeLa nuclei in the presence of 750 mM NaCl [21] by QINESIn. Biological replicates are shown in Suppl. Fig. 1A. (B) comparison of H2A.Z elution curves of H2A.Z in DKO/ΔC and DKO/Z1 cells. Biological replicates are shown in Suppl. Fig. 1F. (C and D) confocal images and line-scans showing the distribution of H2A.Z and H3 in the intercalator-untreated HeLa nuclei (C), and in nuclei treated with 100 µg/ml EBr (D), both in the presence of 750 mM NaCl. (E and F) intercalator elution curves of H2A.Z and H3 containing nucleosomes in HeLa nuclei (E), and of H2A.Z nucleosomes in DKO/ΔC and DKO/Z1 cells (F), with or without nickase pretreatment of the agarose-embedded nuclei. Biological replicate of the experiment in panel (E) is shown in Suppl. Fig. 2C error bars of the histone elution curves represent the SEM of ~600 G1 nuclei measured by LSC in a single experiment.

In the QINESIn assay [21], the agarose-embedded nuclei prepared by nonionic detergent treatment of the embedded, live cells, also referred to as permeabilized nuclei, are exposed to a concentration series of nucleosome-destabilizing agents. In the current experiments, each well of the Ibidi chambers was treated with a different concentration of intercalators, with ethidium bromide (EBr) in the case of Figure 1. The H2A.Z molecules retained in the nuclei in the different wells were then detected by indirect immunolabeling using an antibody recognizing the histone variant in relatively stable and unstable nucleosomes alike (termed ZAbA in [25]; see Materials and Methods). The immunofluorescence of ~1000–1500 nuclei/well is recorded by a laser scanning cytometer (LSC) and the mean fluorescence intensities of the G1-phase nuclei gated based on their DNA content are represented as the points of the elution profiles. As an internal control, the GFP signal of these H3-GFP-expressor HeLa nuclei was used; H3K27me3 or H3K9me3 immunofluorescence were applied for similar purposes in other experiments, as noted there.

As Figure 1 and Suppl. Fig. S1A and B show, the H2A.Z nucleosomes stood out from among the nucleosomes compared by applying EBr as the nucleosome destabilizing agent (in the presence of 750 mM salt, based on the titration performed in [21]). A major intercalator-resistant subpopulation of H2A.Z remained in the nuclei, in contrast with the conduct of H2A or H2A.X- nucleosomes (Figure 1(A), Suppl. Fig. S1A, B), independently of cell cycle phases (Suppl. Fig. S1C, D) and H2A.Z isotypes (Suppl. Fig. S1E) as measured in HeLa cells. As Figure 1(B) and Suppl. Fig. S1F demonstrate, the sensitivity of the H2A.Z-nucleosomes to the intercalator exhibited a marked dependence on the histone’s C-terminal tail, according to the experiments performed in DT40 cell lines expressing the full length or the truncated version of H2A.Z. In the latter case, the last nine amino acids of the H2A.Z C-terminal tail were deleted. Unlike in salt elution, where H2A.Z and H3 behaved very similarly, about 50% of all H2A.Z recognized by the antibody has dissociated while H3 stayed chromatin-associated and, at high EBr concentrations, the majority of H3 was eluted leaving H2A.Z behind in the nucleus of HeLa cells (Figure 1(A), Suppl. Fig. S1A). Thus, H2A.Z and bulk H3 levels, when measured simultaneously in this cell-by-cell assay, changed independently from each other. These data suggest that H2A.Z is not bound to bulk H3, arguing against a possible interpretation of the salt elution results [25]. On the other hand, H3K27me3 and H3K9me3 histones, characteristic for the facultative or constitutive heterochromatin, respectively, showed higher intercalator resistance than bulk H3 measured in HeLa cell nuclei, with a large fraction of H3K27me3 and H3K9me3-nucleosomes remaining chromatin-bound (Suppl. Fig. 1 G, H), suggesting that the EBr resistant H2A.Z nucleosomes may be associated with heterochromatin. In line with this interpretation, the scattered distribution of H2A.Z in the intercalator-untreated nuclei (Figure 1(C)) turned into a perinuclear-perinucleolar topography (Figure 1(D)), and when the composition of the EBr resistant protein fraction of these HeLa cell nuclei was analyzed by mass spectrometry (MS) (Suppl. Fig. 2A, B), several proteins known to be part of heterochromatin were detected.

Intriguingly, upon the pretreatment of the permeabilized nuclei of agarose embedded HeLa cells with a nickase (a restriction enzyme that cleaves only one strand of the double helix) which has many recognition sites in the genomic DNA, less of the full-length variant histone remained chromatin-attached upon intercalator treatment (Figure 1(E); Suppl. Fig. S2 C). The conditions of nickase treatment were such that the incidence of nicks in these circumstances was about 1 nick/10 kb DNA [22]. These observations, together with those of Suppl. Fig. S1G, H demonstrate that the integrity of H2A.Z-containing heterochromatin is sensitive to nickase treatment. The observed sensitivity of H2A.Z-nucleosome stability to topological relaxation is in striking contrast with the lack of any effect of histone acetylation and H2A.Z isotype composition; similarly to the results of the salt elution experiments [25], the stability of the H2A.Z-nucleosomes to intercalators was not affected by acetylation, as it remained unaltered upon treatment of HeLa cells with the histone deacetylase inhibitor trichostatin A (TSA; Suppl. Fig. 2D, E), or in DT40 cells expressing a mutated H2A.Z devoid of N-terminal acetylations [27] (Suppl. Fig. S2D). The antibody used to detect H2A.Z binds with a somewhat higher affinity to the EBr-resistant H2A.Z subpopulation than to H2A.Z before intercalator exposure (Suppl. Fig. 3A) confirming that the antibody specifically recognizes H2A.Z even in the EBr-resistant fraction as measured in HeLa nuclei. The EBr resistant H2A.Z nucleosomes could be eluted by > 1 M NaCl exhibiting similar salt sensitivity to that of the EBr-untreated H2A.Z nucleosomes (Suppl. Fig. S3B, C), suggesting that they persist in their native nucleosomal context.

**Figure 2.**
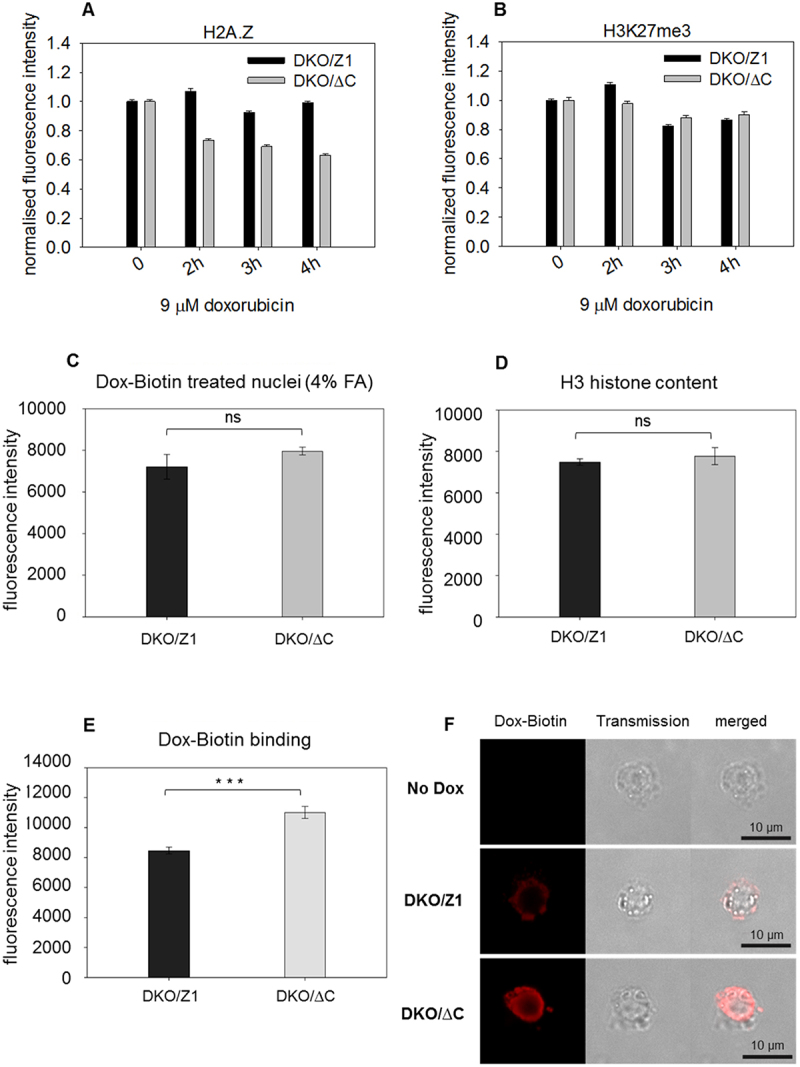
Comparison of histone eviction by Dox and its binding in DKO/ΔC and DKO/Z1 nuclei. (A) H2A.Z, and (B) H3K27me3 containing nucleosomes retained in DKO/ΔC and DKO/Z1 nuclei, after exposure to Dox, measured in the same co-labeled sample as each other’s internal control. Error bars represent the SEM of ~600 G1 nuclei measured by LSC. The result of a biological replicate is shown on Suppl. Fig. 3D, E. (C) Dox-biotin binding in the nuclei of the cell line pair when the cells were fixed with 4% paraformaldehyde prior to Dox-biotin treatment. There is no significant difference between DKO/Z1 and DKO/ΔC. (*p*= 0.1050) (D) comparison of the amount of immunolabeled H3 in the two nuclei. There is no significant difference between DKO/Z1 and DKO/ΔC. (*p*= 0.3227) (E) comparison of the binding of Dox-biotin to DNA in native (unfixed) DKO/ΔC and DKO/Z1 nuclei. The columns show the average immunofluorescence of nuclei measured by LSC, using labeled anti-biotin. Bar charts show the mean fluorescence intensities, error bars represent the SD, for 3 biological replicates. The difference between DKO/Z1 and DKO/ΔC is statistically significant. (*** *p*=0.0007) (F) Representative confocal images showing Dox-biotin in the nuclei of the two cells. No Dox: intercalator-untreated DKO/Z1 nuclei. For statistical analysis unpaired t test was used (in panels C, D and E).

Another intercalator, Doxorubicin (Dox; an anthracyclin used in cancer chemotherapy [28]), which requires no added salt for nucleosome eviction in our previous experience [21], was also tested. Similarly to the effect of EBr, exposure to Dox gave rise to a higher degree of H2A.Z-release in the case of nuclei prepared from DT40 cells expressing the truncated histone as compared to nuclei derived from the control cells, while the H3K27me3-marked facultative heterochromatin behaved very similarly in the two DT40 nuclei (Figure 2(A,B), Suppl. Fig. S3D, E). We also examined if the higher degree of H2A.Z elution in nuclei containing tail-less H2A.Z is paralleled by a higher degree of DNA binding of the intercalator. For this purpose, biotin-labeled doxorubicin [29] was applied. We have shown using fixed cells that the intercalator-untreated DT40 cells expressing full-length or truncated H2A.Z have the same amount of nucleosome-free DNA, hence the same nucleosome content (Figure 2(C,D)). On the other hand, comparison of native nuclei of the cell line pair revealed that more Dox-biotin binds to the DNA of the DKO/∆C nuclei (Figure 2(E,F)). These data, obtained independently from the histone elution measurements, further confirm that the truncated H2A.Z-containing nucleosomes are more readily evicted upon exposure to an intercalator as compared to the nucleosomes of the control cells.

**Figure 3.**
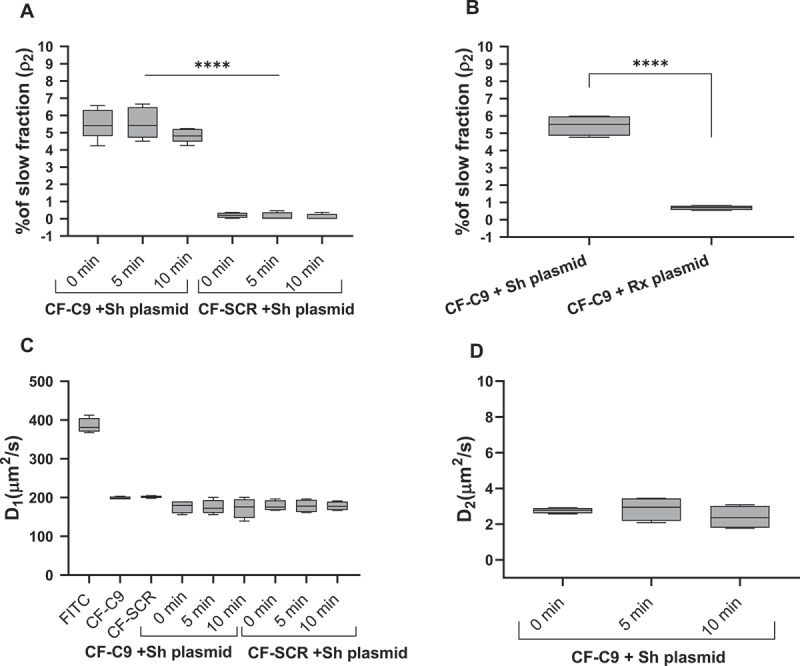
CF-C9 binding to plasmid DNA as measured by FCS. (A) slow fraction of CF-C9 and CF-SCR added to superhelical (Sh) plasmid DNA. The peptides were incubated with the plasmid for 0 (measured promptly after addition), 5 and 10 min. The slow fraction (ρ_2_) was calculated in the autocorrelation function (ACF; see materials and methods) fits and presented as a box-and-whisker plot. The difference between CF-C9+Sh and CF-SCR+Sh plasmids is statistically significant (*** *p*=0.0001). For statistical analysis, one-way ANOVA test was used. (B) comparison of the slow fraction of CF-C9 when the peptide was incubated with superhelical (Sh) or relaxed (rx) plasmid DNA. The difference between CF-C9+Sh and CF-C9+Rx plasmids is statistically significant (*** *p*=0.0001). For statistical analyses unpaired t test was used. (C) diffusion coefficient D_1_ corresponds to the faster-diffusing component, and (D) D_2_ to the slower-diffusing component of panel A. Box-and-whisker plots in panel A and B were created from data of four independent replicates. C and D show the diffusion coefficients measured in one experiment out of the four replicates.

The significant differences in the conduct of H2A.Z-nucleosomes in DKO/Z1 and DKO/∆C nuclei in intercalator elution and intercalator binding, as well as in their response to nickase treatment, raised the possibility that the histone tail may bind to DNA in a superhelicity-dependent fashion. Indeed, when binding of the carboxyfluorescein-labeled tail peptide (CF-C9 [25]) to superhelical and relaxed, circular plasmid DNA was compared in FCS measurements, the supercoiled plasmid was the preferred target of CF-C9 but not of the control peptide (Figure 3(A,B)). The diffusion constant of the fluorescent dye itself exceeded those of the dye-labeled peptides (Figure 3(C)) which in turn was even lower in the case of the slow component (Figure 3(D)), as expected.

**Figure 4.**
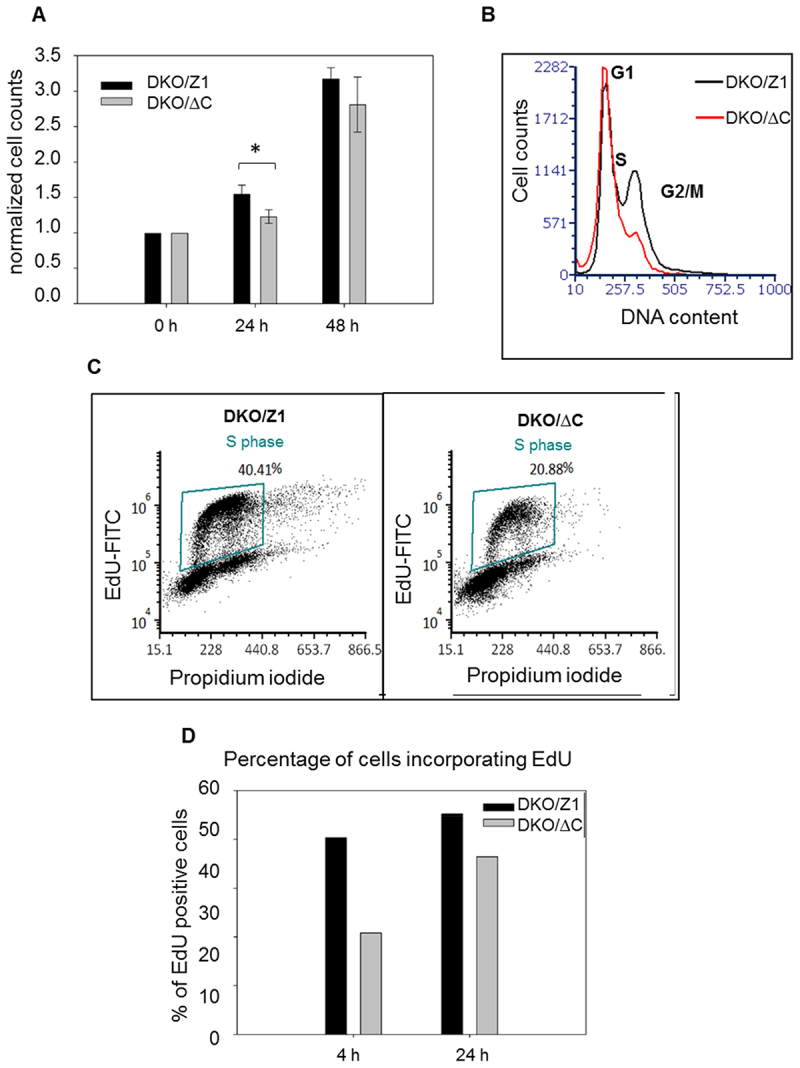
The effect of the absence of the C-terminal H2A.Z tail on cell proliferation. (A) normalized cell counts of DKO/ΔC and DKO/Z1 cells at different time points upon culturing. Bars show SD of three independent experiments. The difference between DKO/Z1 and DKO/ΔC is statistically significant. (* *p*=0.0234) (B) flow-cytometric DNA distribution histograms of the cell pair taken on day 0 of culturing. (C) comparison cell proliferation using the EdU incorporation assay 4h after splitting the cells. (D) percentage of cells incorporating EdU (using the gates shown in panel C) after 4 h and 24h of cell culturing prior to EdU addition in a representative experiment (see also the results of an independent biological experiment in Suppl. Fig 4).

The measured diffusion constant of the slow component (D_2_) representing the complex of CF-C9 and the superhelical plasmid is in agreement with data available in the literature [30]. The D value of the center of mass of superhelical plasmids was found to be proportional to the −2.2th power of the plasmid length [30]; based on this model and on the D value of a 5.9 kb supercoiled plasmid determined by differential dynamic microscopy (0.44 µm^2^/s), the predicted D value of our 4.48 kb supercoiled plasmid would be 0.81 µm^2^/s. The FCS-determined D_2_ of the slow component was ~2–3 µm^2^/s (Figure 3(D)). Taking into account that the plasmid has internal motion superimposed on the motion of the center of mass thereby increasing the apparent D, this value can be attributed to the motion of the DNA-bound CF-C9. We conclude that the CF-labeled nonapeptide representing the C-terminal end of H2A.Z preferentially binds to the negatively superhelical plasmid DNA as compared with the relaxed plasmid, suggesting that the C-terminus of the native protein may also engage with the DNA in chromatin in a superhelicity-dependent manner. This binding, or the lack of it when the tail is bound to a reader protein, may have biological significance since the proliferative capacity of the DT40 cells expressing C-terminally truncated H2A.Z exhibit a considerable growth defect relative to the control cells, as shown in Figure 4 and Suppl. Fig. S4.

Challenging nucleosome structure by changing the superhelicity of the DNA wound around, as well as interconnecting them, rather than perturbing ionic and hydrogen bonds by salt, offers an independent approach to characterize their stability [21]. Importantly, alterations of superhelicity occur also *in vivo* upon processive enzymatic processes of the nucleus, thus the differences in stability observed through the spectacles of this assay may be of direct biological significance. By exploring how H2A.Z-nucleosomes behave when exposed to increasing concentrations of two different intercalators we observed marked differences in comparison with nucleosomes containing canonical histones or H2A.X (Figure 1(A), Suppl. Fig. 1A, B). The different conduct of full length and truncated H2A.Z-containing nucleosomes upon exposure to intercalators, with or without nickase pretreatment (Figure 1(B,E,F); Figure 2(A,E,F); Suppl. Fig. 1F, Suppl. Fig. 2C) supported also by the FCS results of Figure 3 confirm the pivotal role of the C-terminal tail of H2A.Z in the stabilization of nucleosomes containing the variant histone, implicating binding of the tail to superhelical, as opposed to relaxed DNA. This observation may also help explain the peculiar chromosomal band-localization of genes down-regulated by C9 introduced into live MEL1617 cells [25]. DNA-binding may involve internucleosomal linker DNA, nucleosomal DNA and DNA in nucleosome-free regions. In the case of the linker DNA, binding may be facilitated by the underwinding of the double-helix in the wake of transcription [31]. For DNA wound around the histone core, segments with a twist resembling that of the superhelical plasmid could be preferably involved [1]. In either case, relaxation of the negative superhelicity established upon nucleosome formation in S phase, by intercalator binding or by nicking, would shift the dynamic equilibrium of nucleosome formation and eviction toward the latter process, i.e. destabilization. In the case of an H2A.Z-nucleosome juxtaposed with a nucleosome-free region, occurring at promoters [23,24], the free DNA in its plectonemic form [2] may bind the tail, while topological relaxation upon the transient DNA breakages at promoters accompanying gene activation [22,32] would yield relatively destabilized promoter-proximal nucleosomes [22]. The association of a large population of H2A.Z with H3K27me3 and H3K9me3-chromatin, suggested by their similarly high EBr-resistance (Suppl. Fig. 1 G, H), is in line with the assumed role of the histone variant in heterochromatin organization [33] and could be relevant also in the context of aberrant heterochromatin spreading [34]. Furthermore, since H2A.Z plays a role in DNA repair, the superhelicity-dependent stability of the nucleosomes containing the histone variant may facilitate its dynamic exchange at the DNA breaks [16,35,36]. The data presented also underline and demonstrate in close-to-native conditions the general role of DNA superhelicity in the regulation of nucleosome stability, as predicted based on scanning force microscopy and FCS studies of nucleosomes reconstituted on plasmids of varying superhelical density [37]. Furthermore, they call the attention to the possibility that superhelicity-dependent interactions between the DNA and certain histone tails may be exploited by the cell for regulatory functions.

## Materials and methods

### Peptides

Peptides representing the C-terminus of H2A.Z (C9) were synthesized and fluorescently labeled with 5(6)-carboxyfluorescein (CF) yielding the peptide H-GKKGQQKTV-Ahx-K(CF)-OH (CF-C9) as described in [25]. As a negative control, a scrambled peptide (SCR) composed of the same nine amino acids as C9, but arranged in a randomized order was created. The sequence was generated by shuffling the residues of C9, and then cross-checked against the UniProtKB peptide database to ensure that it did not match any known peptide. The final control peptide sequence is: H-KQGTGKVQK-OH.

### Plasmid

The plasmid DNA (pCMV-EGFP-4X, 4479 bp), provided by Dr. Katalin Tóth (DKFZ, Heidelberg), used for assessing the DNA-binding of CF-C9 and CF-SCR by fluorescence correlation spectroscopy (FCS) analysis, was introduced into Escherichia coli DH5α by heat shock and selected on LB agar plates containing 100 μg/ml kanamycin.

### Cell culture

H2A.Z.1 expressing DKO DT40 cells (DKO/Z1), and DKO DT40 expressing C-terminally truncated H2A.Z.1 (DKO/ΔC), were provided by Dr. Masahiko Harata (Sendai, Japan) [12]. These cells were maintained in DMEM supplemented with 2% chicken serum, 8% fetal calf serum (FCS), 2 mM L-glutamine, 100 μg/ml streptomycin, and 100 U/ml penicillin at 38.9°C in 5% CO₂ atmosphere.

### Cell cycle analyses

DKO/Z1 and DKO/ΔC cells, cultured in 6-well plates at 250,000 cells/well density, were harvested by centrifugation at 1500 rpm for 5 min at RT, then the cell pellets were washed with ice-cold PBS and fixed with 500 μl of 70% ethanol added gradually to reduce clumping. After fixation on ice for 1 hour, the cells were washed twice with ice-cold PBS and treated with RNaseA (100 µg/ml) at RT for 30 min, followed by staining with propidium iodide (PI, 5 µg/ml) on ice for 10 min. The stained samples were washed with PBS, resuspended in 200 µl of PBS, and analyzed using a Novocyte flow cytometer. Data were processed using the FCS Express 6 software.

### EdU cell proliferation assay

The manufacturer’s protocol for the Invitrogen Click-iT™ Plus EdU Flow Cytometry Assay Kit (#C10632) was followed. Briefly, DKO/Z1 and DKO/ΔC cells were seeded in 6-well plates with complete media and cultured for 4, 24, or 48 hours. EdU was applied drop-wise to each well, followed by 1-hour incubation at 37°C. The cells were then washed with 1% BSA/PBS, fixed, washed again, and resuspended in 100 µl permeabilization buffer for 15 min. The reaction cocktail was added to the samples in a volume of 495 µl and incubated at room temperature in the dark for 30 min. Finally, the cells were washed, treated with RNase A, stained with PI and analyzed as above.

### Embedding live cells into low melting point agarose and permeabilization

Embedding of cells into 8-well microscopic chambers (Ibidi, Martinsried, Germany) coated with 1% (m/V) low melting point (LMP) agarose was as described earlier [21]. Briefly, after washing with ice-cold PBS/EDTA three times for 3 min each, the cells were permeabilized by treatment with 500 μl of ice-cold 1% (V/V) Triton X-100 dissolved in PBS/EDTA (5 mM EDTA in PBS) twice for 10 min each.

### Treatment of the permeabilized nuclei of agarose embedded cells with intercalators, immunostaining

After permeabilization, nuclei were washed five times with 500 μl ice cold PBS/EDTA for 3 min each. Ethidium bromide (EBr) treatment was performed using concentrations ranging from 0 to 100 μg/ml in PBS/EDTA containing 750 mM salt for 1 hour on ice followed by washing three times with 500 μl ice cold PBS/EDTA for 10 min each. H2A.Z was stained by overnight incubation at 4°C with the primary antibody (anti-H2A.Z Rabbit Polyclonal Antibody, Abcam ab97966; anti-H2A Rabbit Polyclonal Antibody, Abcam ab18255; anti-H2A.X Rabbit Polyclonal Antibody, Abcam ab20669; anti-H3 Rabbit Polyclonal Antibody, Abcam ab1791; anti-H3K27me3 mouse monoclonal antibody and H3K9me3 mouse monoclonal antibody, both provided by Prof. Hiroshi Kimura [25] diluted 1:800 in 1% BSA in PBS/EDTA. After four sequential washes (quick, 10, 30, and 60 min) with 500 μl ice cold PBS/EDTA, nuclei were incubated at 4°C in the dark with a secondary antibody (Alexa Fluor 488 Goat Anti-Rabbit, Invitrogen A11008 or Alexa Fluor 647 Goat Anti-Mouse, Invitrogen 21,235) diluted 1:800 in 1% BSA in PBS/EDTA. When biotin-labeled Doxorubicin (Dox-biotin) was used as an intercalator, the washed nuclei were incubated with 200 μl of 1 μM Dox-Biotin dissolved in PBS/EDTA (from Zutao Yu, Cambridge University, UK) for 2 hours on ice in the dark, followed by three washes with 500 μl ice cold PBS/EDTA (10 min each). Samples were stained overnight at 4°C in the dark with 150 μl of the primary antibody (mouse anti-biotin, Sigma B7653), and after four washes (as above), were incubated with the secondary antibody (Alexa Fluor 647 goat anti-mouse, Invitrogen A21235) under the same conditions.

Samples were fixed in 200 μl of 1% formaldehyde overnight at 4°C in the dark. (Fixation was performed after intercalator treatment and immunostaining, i.e. histone elution was not affected by formaldehyde crosslinking and antigen epitopes recognized by the antibodies were not modified before labeling.) The next day, formaldehyde was removed, and the samples were washed three times with 500 μl ice cold PBS/EDTA for 5 min each before staining with 12.5 μg/ml (in case of EBr) for 1 hour on ice. After three additional washes with 500 μl ice cold PBS/EDTA for 3 min each, fluorescence intensity distributions were recorded using an iCys Laser Scanning Cytometer (LSC). The data were analyzed using the iCys0.7 software, and statistical analyses were performed in GraphPad Prism V8.2.1.

### Nickase treatment of nuclei

Cells were embedded into agarose and permeabilized as described above. The frequent cutter Nt.CviPII nickase (recognition site: CCD; New England Biolabs Inc., Ipswich, Massachusetts, USA) was applied after the washing steps following permeabilization. Before digestion, the samples were equilibrated with nickase buffer (10 mM Tris-HCl pH 8.0, 50 mM NaCl, 10 mM MgCl_2_, 1 mg/ml BSA) by washing three times with 500 µl of the buffer solutions. Nickase treatment was performed in 300 µl nickase buffer for 30 min at 37°C, using the enzyme at a final concentration of 0.5 U/ml. After enzymatic treatment, the samples were washed with 500 μl ice cold PBS/EDTA three times, for 3 min.

### Fluorescence correlation spectroscopy (FCS)

The native plasmid DNA was either nicked or linearized using 1 U of Nb.BsmI (ER2051) or 1 U of EcoRI (ER0275), respectively, in a 20 μl reaction volume, for 1 hour at 37°C. Both enzymes were from Thermo Scientific, Waltham, Massachusetts, USA. Equal amounts of supercoiled, nicked and linearized plasmid DNA were mixed and loaded into wells of a 1% agarose gel. The bands were cut from the gel and the DNA was isolated and purified using a Promega kit (Wizard® Plus SV Minipreps DNA Purification Systems, A1460).

In FCS, the fluorescence intensity fluctuations of molecules diffusing across the sub-femtoliter detection volume illuminated by a focused laser beam is measured in a confocal arrangement. The temporal autocorrelation function (ACF) of the fluorescence intensity gives information about the mobility, absolute concentration and aggregation state as well as the photophysical properties of the molecules [38]. FCS was used to assess the binding of CF-C9 and CF-SCR peptides to superhelical or relaxed plasmid DNA (pCMV-EGFP-4X) based on the FCS-derived mobility of the peptides/peptide-DNA complexes.

For sample preparation, all solutions were kept on ice. A freshly prepared 1 M stock of the antioxidant vitamin C was made by dissolving 176 mg of vitamin C powder in 1 ml of deionized water and vortexing until fully dissolved. For calibration, 20 nM of Alexa Fluor 488 (A488) was made from a 10 µM stock, which was centrifuged at 14,000 rpm for 10 min at 4°C, then dissolved in ice-cold TE buffer (10 mM Tris, 0.1 mM EDTA, pH 7.4). 400 nM of the carboxyfluorescein labeled C9 peptides CF-C9 and CF-SCR (scrambled) were made from 2 mM stock, which were diluted stepwise in ice-cold PBS under thorough vortexing, then centrifuged at 14,000 rpm for 10 min at 4°C. A base solution was prepared by mixing 3.992 ml of TE buffer (pH 7.4, 4 µl of 1 M vitamin C (1 mM), and 4 µl of 10% NP-40 detergent (0.01%). FITC (control) were measured by FCS at a final concentration of 20 nM (in 10 mM Tris-EDTA buffer, pH 7.4), while the peptides CF-C9 and CF-SCR were added at 40 nM in PBS. The peptides were either measured alone or in combination with 10 µg/ml plasmid DNA (pCMV-EGFP-4X) in a supercoiled form or relaxed by nickase using Nb.BsmI (Thermo Scientific ER2051, Waltham, Massachusetts, USA.). FCS measurements were performed using 8-well microscopic chambered slide (Ibidi, Martinsried, Germany) with a sample volume of 200 µl at room temperature (22.5 °C), using a Carl Zeiss LSM 880 confocal microscope (Carl Zeiss, Jena, Germany), equipped with a 60× water immersion objective and a photon counting detector. Fluorescence of the CF-tagged peptide was excited by the 488 nm laser line and its emission was detected between 500–550 nm.

### Evaluation of raw FCS data

FCS measurements consisted of 5 × 20 s runs, and each sample was measured at least three times at each condition. FCS data were evaluated by using the QuickFit3 software ((JW. Krieger, J. Langowski (2015): QuickFit 3.0 (status: beta, compiled: compiled: 5 January 2015, SVN: 3695): A data evaluation application for biophysics, [web page] http://www.dkfz.de/Macromol/quickfit/[Accessed on 5 January 2015]. Autocorrelation functions from each run were inspected, and those displaying artifacts due to large fluctuations caused by aggregates were excluded. The remaining runs were averaged, and the resulting correlation curve was fitted to different models using a simulated annealing algorithm with box constraints weighted by the standard deviations of the runs. We tested normal (free Brownian) and anomalous diffusion models with a two-component normal diffusion model for measurements of Plasmid + CF-C9 (or CF-SCR) and a one-component model for the CF-C9 (or CF-SCR) alone to fit ACFs.

Each model included a triplet term:

(1) $$G\left(\tau \right)=\frac{1-T+Te^{-\tau \;/\;\tau_{\mathrm{tr}}}}{1-T}G_{\mathrm{diff}}\left(\tau \right)$$

where,

(2) $$G_{\mathrm{diff}}^{no\mathrm{rmal}}\left(\tau \right)=\frac{1}{N}\left[\rho_{1}{\left(1+\frac{\tau }{\tau_{1}}\right)}^{-1}{\left(1+\frac{\tau }{S^{2}\tau_{1}}\right)}^{-1/2}+\rho_{2}{\left(1+\frac{\tau }{\tau_{2}}\right)}^{-1}{\left(1+\frac{\tau }{S^{2}\tau_{2}}\right)}^{-1/2}\right]$$

*N* denotes the average number of diffusing fluorescent molecules present in the detection volume, *τ* is the lag time, *T* is the equilibrium mole fraction of fluorophores in triplet state, and *τ*_*tr*_ is the triplet correlation time [39,40].

In the model, we assumed one or two distinct diffusing species: a fast population with a fraction of *ρ*_*1*_, a diffusion time of *τ*_*1*_ and a slow one with a fraction of *ρ*_*2*_ and a diffusion time of *τ*_*2*_; *ρ*_*2*_ equals 1-*ρ*_*1*_. *S* is the ratio of the axial and longitudinal diameters of the ellipsoid-shaped confocal detection volume, defined by the properties of the microscope. *S* was determined before each measurement by fitting the ACFs of the 20 nM fluorescein dye solution (in 10 mM Tris-EDTA buffer, pH 7.4).

The diffusion coefficients of the fast and slow components were calculated by:

(3) $$D_{i}={\omega_{\mathrm{xy}}}^{2}/4\tau_{i}$$

where *ω*_*xy*_ is the lateral radius of the detection volume. *ω*_*xy*_ was calculated from the measured diffusion time of 20 nM fluorescein solution dye as follows:

(4) $$\omega_{\mathrm{xy}}=\sqrt{4D\tau_{D}}$$

where *τ*_*D*_ is the diffusion time of the dye, and *D* is its diffusion coefficient taken from the literature (425 μm^2^/second at 25°C) [41].

### Confocal laser scanning microscopy

Confocal images were taken using Nikon A1 laser scanning confocal microscope (Nikon, Tokyo, Japan), equipped with a Plan Apo 60 × NA 1.27 water immersion objective. Alexa 488 was excited by the 488 nm laser. Alexa 647 and PI were excited by the 633 nm and 543 nm lasers, respectively. Images were analyzed using Fiji ImageJ.

### Laser scanning cytometry (LSC)

Automated microscopy imaging was done using an iCys laser scanning cytometer (Research Imaging Cytometer; CompuCyte, Westwood, Massachusetts, USA). The instrument is based on an Olympus IX-71 inverted microscope equipped with four lasers, photodiodes (detecting light loss and scatter) and four photomultiplier tubes (PMTs). The 488 nm Argon ion laser was used to excite Alexa 488 and PI and the 633 nm HeNe laser was applied in the case of Alexa 633. Fluorescence signals were collected via an UPlan FI 20× NA 0.5 objective. Alexa 488 fluorescence was detected through a 530/30 nm bandpass filter, while Alexa 647 and PI were detected through a 650/LP filter. Data analysis was performed using the iCys 7.0 software, and graphs were prepared using SigmaPlot 11.0.

### Statistical analysis

The data from each experiment are shown as the mean and standard deviation (SD) of n ≥ 3 biologically independent experiments. Data were plotted in SigmaPlot 11.0 and statistical analyses were performed with GraphPad Prism V8.2.1 using one-way ANOVA or unpaired Student’s t-tests, as indicated in the legends. Exact numbers of the *p* values of statistical significance are indicated in the legends.

## Supplementary Material

Supplemental Material

## Data Availability

All data that support the findings of this study are shared by the corresponding author upon request.

## Abbreviations

FCS: fluorescence correlation spectroscopy
CF-C9: carboxyfluorescein-tagged
H2A.Z: tail nonapeptide
Dox-Biotin: biotin-labeled Doxorubicin
TSS: transcription start site
PTM: posttranslational modification
EBr: ethidium bromide
