# Supplementary material for: H2A.Z-nucleosomes are stabilized by the superhelicity-dependent DNA binding of the C-terminal tail of the histone variant
Source: Nucleus. 2025 Sep 9;16(1):2557113. doi: 10.1080/19491034.2025.2557113 (PMC12427435; doi:10.1080/19491034.2025.2557113)
Supplement: Supplemental Material [file KNCL_A_2557113_SM9985.docx]

**Supplementary Figures**

**
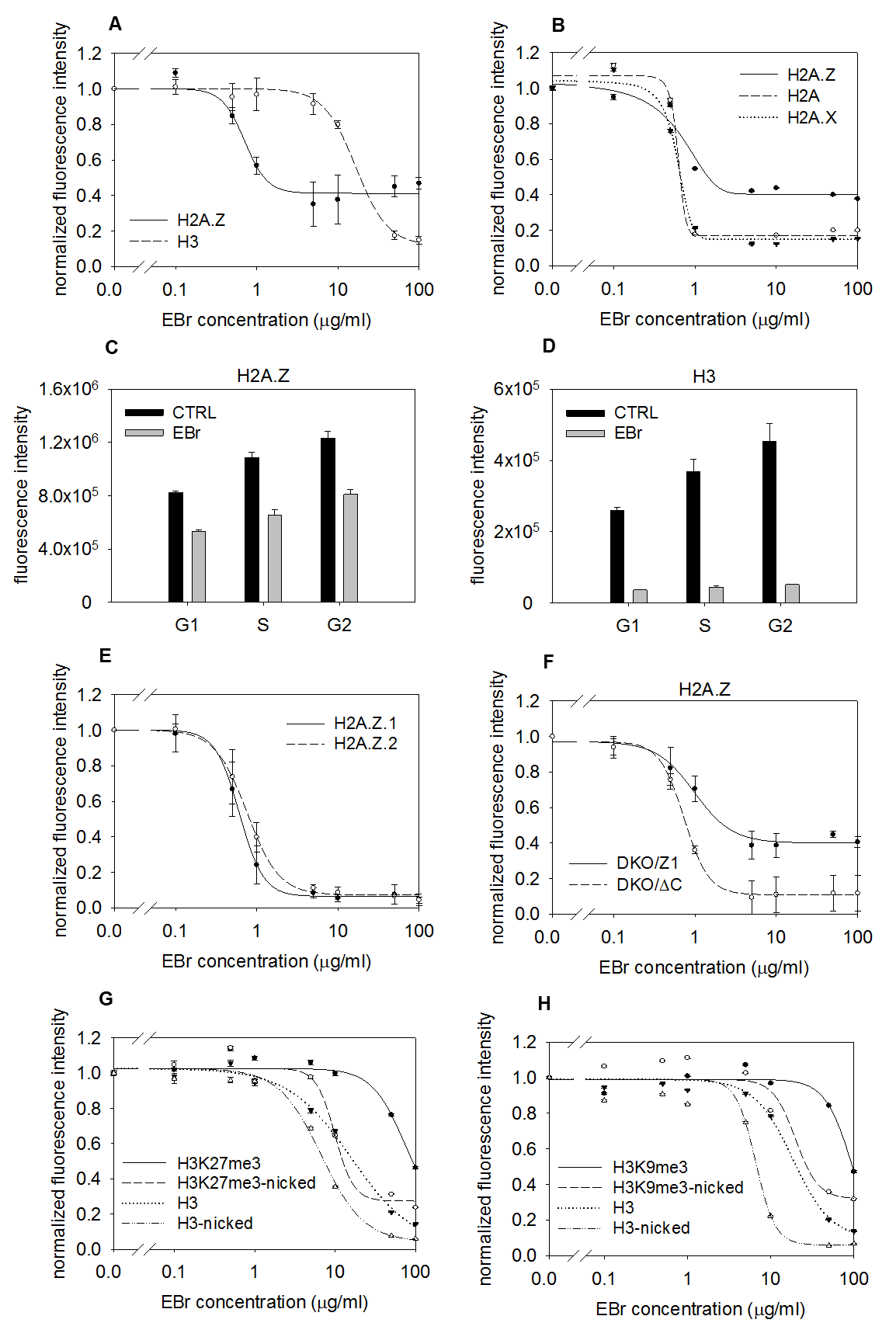
**

**Suppl. Fig. 1.**

**(A)** EBr elution profile of H2A.Z compared to GFP-tagged H3 used as an internal control in HeLa cell nuclei measured by QINESIn. The curves show the means of the histone content of ~600 G1 phase nuclei gated based on their DNA content. Averages and SD values of three independent biological experiments are shown. Half-maximum of the fitted H2A.Z curve: EC_50_= 0.7 **(B)** Comparison of the intercalator elution profiles of H2A, H2A.X, H2A.Z in HeLa nuclei, using EBr in the presence of 750 mM NaCl measured by QINESIn. **(C and D)** Comparison of the intercalator resistance of nucleosomal H2A.Z **(C)** and H3-GFP **(D)** in the different phases of the cell-cycle in HeLa nuclei, using 100 µg/ml EBr. The height of the bars represents the mean fluorescence intensity, the error bars represent the SEM of ~600 G1 phase cells. **(E)** EBr elution curves of H2A.Z1-CFP and H2A.Z2-YFP tagged on their C-terminus, measured in transiently transfected HeLa nuclei ([25](#_ENREF_25)). Averages and SD values of three independent biological experiments are shown. **(F)** EBr elution profiles of H2A.Z in H2A.Z.1ΔC (DKO/ΔC) and H2A.Z.1 (DKO/Z1) expressor DKO DT40 cells. The elution curves refer to G1 phase nuclei gated according to their DNA fluorescence intensity distribution of ~600 nuclei measured by QINESIn. Averages and SD values of three independent biological experiments are shown. Half-maxima of the fitted H2A.Z curve in the case of DKO/Z1 cells: EC_50_= 0.9) **(G)** EBr elution profile of H3K27me3 compared to GFP-tagged H3 used as an internal control, in HeLa nuclei. The error bars represent the SEM of ~600 G1 phase cells. **(H)** EBr elution profiles of H3K9me3 compared to GFP-tagged H3 used as an internal control, in HeLa nuclei. The error bars represent the SEM of ~600 G1 phase nuclei.


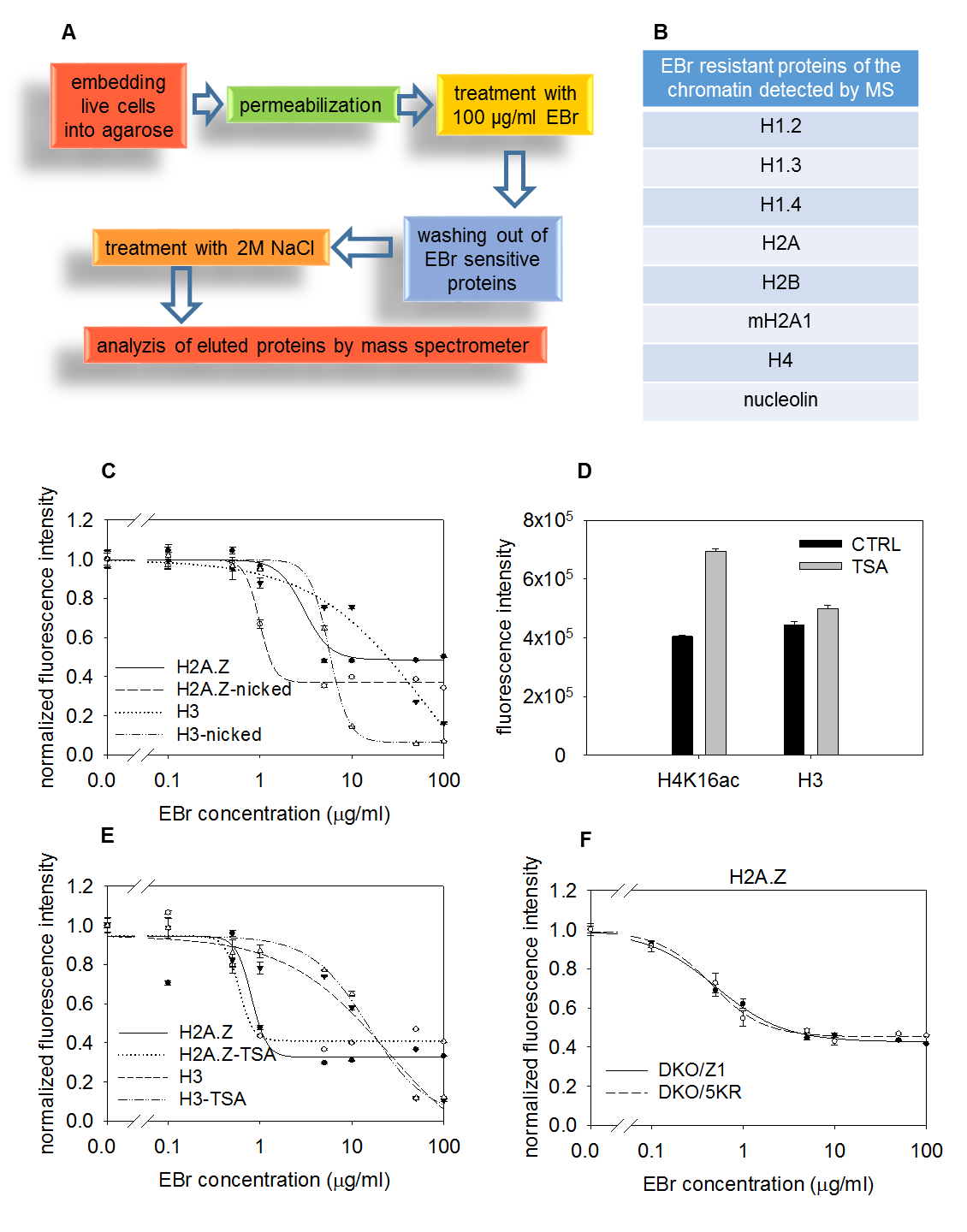


**Suppl. Fig. 2.**

**(A)** Flow chart EBr elution combined with mass spectrometry (MS). Proteins resistant to 100 µg/ml EBr treatment were detected by MS after high salt (2 M) elution, as [[25](#_ENREF_25)] described in [[25](#_ENREF_25)]. **(B)** Some of the proteins identified by MS in the intercalator resistant fraction known to be chromatin associated are listed in the table. **(C**) EBr elution profiles of H2A.Z compared to GFP-tagged H3 used as an internal control, before and after 0.5 U/ml nickase treatment (see Materials and Methods) of permeabilized HeLa nuclei. The elution curves refer to G1 phase nuclei gated according to their DNA fluorescence intensity distribution. The error bars represent the SEM of ~600 G1 nuclei measured by LSC. **(D)** Effect of TSA treatment (100 nM for 48h) of HeLa cells on the level of H4K16ac compared to the amount of GFP-tagged H3 used as an internal control. The mean fluorescence intensity of ~600 G1 phase nuclei gated according to their DNA content and the error bars representing the SEM values are shown. **(E)** EBr elution profiles of H2A.Z compared to GFP-tagged H3 used as an internal control, with and without 100 nM TSA pretreatment of HeLa cells for 48 hours. **(F)** EBr elution profile of H2A.Z nucleosomes in H2A.Z.1-5KR (DKO/5KR) and H2A.Z.1 (DKO/Z1) expressor DKO DT40 cells. In the H2A.Z.1-5KR mutant, 5 acetylatable lysines on the H2A.Z N-terminus were changed to arginines [[27](#_ENREF_27)].


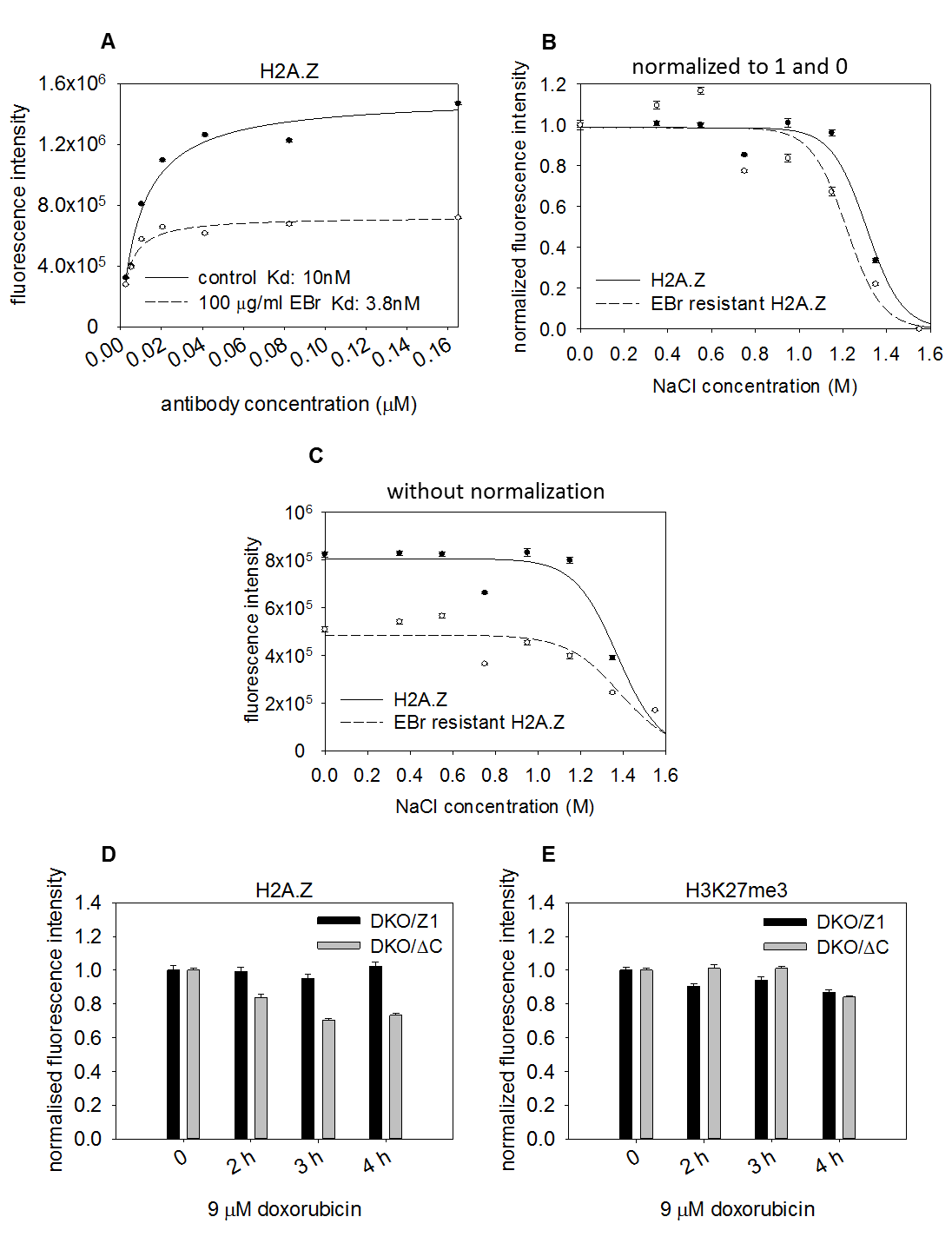


**Suppl. Fig. 3.**

**(A)** Determination of the dissociation constants (K_d_) of the anti-H2A.Z antibody (Abcam. ab97966) used throughout the experiments in nuclei containing total H2A.Z (control) and nuclei with only the intercalator-resistant H2A.Z species. The intercalator sensitive H2A.Z subpopulation was evicted by treatment with 100 µg/ml EBr. The error bars represent the SEM of ~600 G1 nuclei measured by LSC. **(B and C)** Salt elution curves of bulk H2A.Z and EBr resistant H2A.Z with **(B)** or without **(C)** normalization of the fluorescence signal to 1 (maximum fluorescence) and 0 (minimum fluorescence). To measure the stability of EBr resistant H2A.Z nucleosomes, the nuclei were pretreated with 100 µg/ml EBr to remove the intercalator-sensitive fraction of H2A.Z before salt elution. The intercalator was washed out from the nuclei at 4ºC, overnight, before the salt elution curves were obtained. The error bars represent the SEM of ~600 G1 nuclei measured by LSC. **(D and E)** H2A.Z **(D)**, and H3K27me3 **(E)** containing nucleosomes retained in DKO/ΔC and DKO/Z1 cells**,** after exposure to Dox, measured in co-labeled samples. Error bars represent the SEM of ~600 G1 nuclei measured by LSC. Biological replicate of Fig. 2A, B.

**
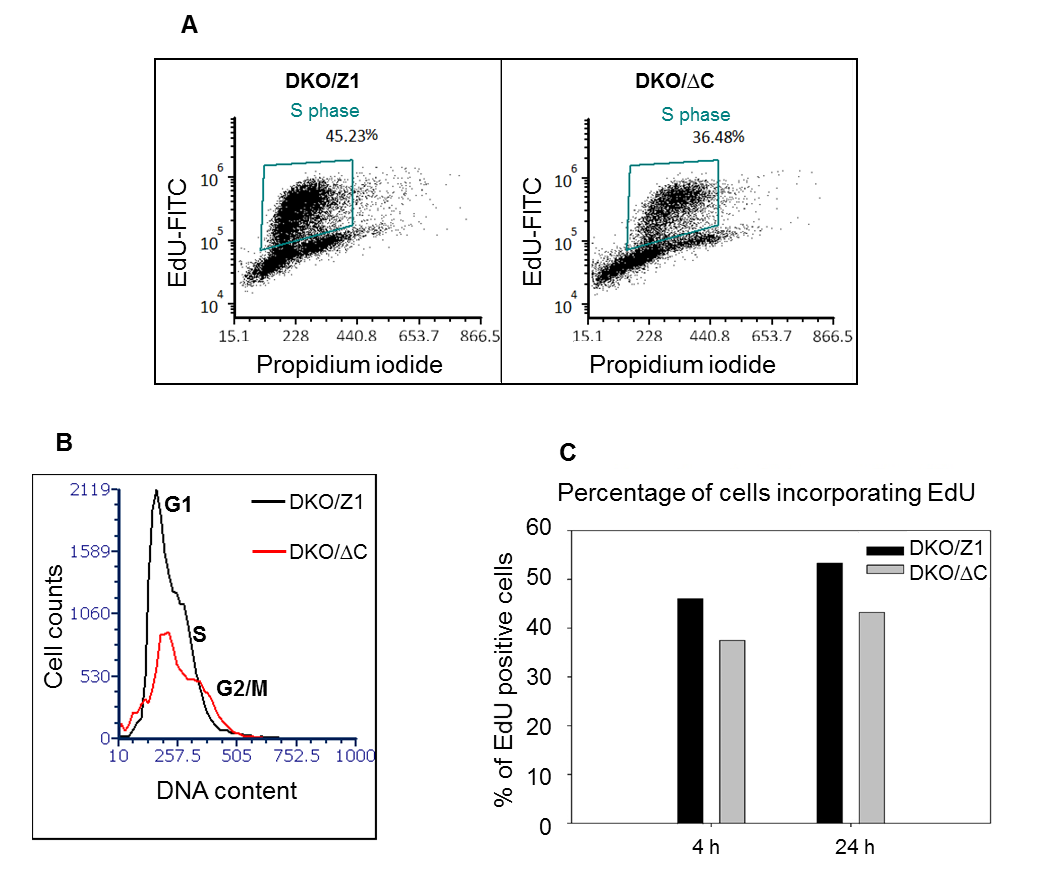
**

**Suppl. Figure 4.**

**(A)** Comparison of cell proliferation of DKO/ΔC and DKO/Z1 cells using the EdU incorporation assay 24h after splitting the cells in an independent biological experiment (see Fig. 4). **(B)** DNA distribution histograms of the cell pair 24h after splitting the cells. **(C)**  Percentage of cells incorporating EdU, after 4h and 24h of cell culturing prior to EdU addition.
